# Beyond Vasospasm: Dual Milrinone and Balloon Angioplasty in Refractory Delayed Cerebral Ischemia Post‐Subarachnoid Hemorrhage

**DOI:** 10.1002/ccr3.72786

**Published:** 2026-05-24

**Authors:** Paul Cardozo Gil, Willy Pozo Soto, Alejandro Sengoku Cadima, Pablo Flores, Jorge Botello Marin, Gustavo Averanga, Richy Hurtado Montaño, Nelson Montalvo Flores

**Affiliations:** ^1^ Clínica de las Américas, Hospital Obrero Nro. 3—Caja Nacional de Salud Santa Cruz Bolivia; ^2^ Hospital Italiano de Buenos Aires Buenos Aires Argentina

**Keywords:** aneurysmal subarachnoid hemorrhage, balloon angioplasty, cerebral vasospasm, delayed cerebral ischemia, milrinone, transcranial Doppler

## Abstract

Cerebral vasospasm is a major complication of aneurysmal subarachnoid hemorrhage and a key contributor to delayed cerebral ischemia, which remains a leading cause of morbidity despite standard therapy with nimodipine and induced hypertension. Up to 30% of patients develop refractory delayed cerebral ischemia, highlighting the need for alternative therapeutic strategies. Milrinone has emerged as a promising option due to its combined vasodilatory and inotropic properties. We report the case of a 42‐year‐old man who developed progressive neurological deterioration following aneurysmal subarachnoid hemorrhage secondary to rupture of an anterior cerebral artery aneurysm. Despite definitive microsurgical clipping and optimal standard management, the patient developed refractory delayed cerebral ischemia with severe vasospasm. A stepwise, physiology‐guided treatment strategy was implemented, including high‐dose intravenous milrinone infusion, intra‐arterial milrinone administration, and rescue balloon angioplasty. This approach resulted in significant angiographic improvement, progressive neurological recovery, and successful ventilatory weaning. This case highlights the value of a multimodal, escalation‐based strategy for the management of refractory delayed cerebral ischemia, supporting the use of milrinone as a potential first‐line rescue therapy and reserving balloon angioplasty for non‐responders. It reinforces the concept of delayed cerebral ischemia as a multifactorial process and underscores the critical role of intensive neuromonitoring in enabling safe and effective therapeutic escalation.

## Key Clinical Message

Delayed cerebral ischemia following aneurysmal subarachnoid hemorrhage should be understood as a multifactorial process rather than an isolated macrovascular phenomenon. A physiology‐guided, stepwise strategy using intravenous and intra‐arterial milrinone, with balloon angioplasty reserved for refractory cases, may contribute to improved cerebral perfusion and neurological outcomes.

## 1 Introduction

Cerebral vasospasm (CVS) is a critical complication of aneurysmal subarachnoid hemorrhage (aSAH), with an angiographic incidence approaching 70% and symptomatic vasospasm occurring in 20%–40% of patients, frequently associated with unfavorable outcomes [1]. Diagnosis relies on angiographic findings or elevated flow velocities detected by transcranial Doppler (TCD), a bedside, noninvasive technique with reported sensitivities approaching 90% [2, 3]. CVS is a major contributor to delayed cerebral ischemia (DCI), a diagnosis of exclusion that requires careful clinical, imaging, and laboratory evaluation to rule out alternative causes of neurological deterioration [4].

Although oral nimodipine remains the only pharmacologic therapy with proven benefit in reducing poor outcomes after aSAH, its effect on angiographic vasospasm is limited. Up to 30% of patients develop DCI progressing to cerebral infarction, and only 20%–35% of survivors achieve good functional recovery (modified Rankin Scale ≤ 2) [5]. These limitations have led to a paradigm shift in which DCI is increasingly recognized as a multifactorial process involving not only large‐vessel vasoconstriction but also microcirculatory dysfunction, neuroinflammation, impaired autoregulation, and endothelial injury.

Milrinone, a phosphodiesterase‐III inhibitor with both cerebral vasodilatory and systemic inotropic properties, has emerged as a promising therapeutic alternative. Emerging evidence suggests that intra‐arterial administration followed by prolonged intravenous infusion may reverse refractory CVS and improve cerebral perfusion [6, 7]. Based on this rationale, we present a case of refractory DCI successfully managed using a stepwise strategy combining intravenous and intra‐arterial milrinone with rescue balloon angioplasty.

## 2 Case History and Examination

A 42‐year‐old man with no significant past medical history presented with progressive neurological deterioration 48 h after endoscopic nasal polyp excision, characterized by somnolence, decreased level of consciousness, and cerebrospinal fluid (CSF) rhinorrhea.

**FIGURE 1:**
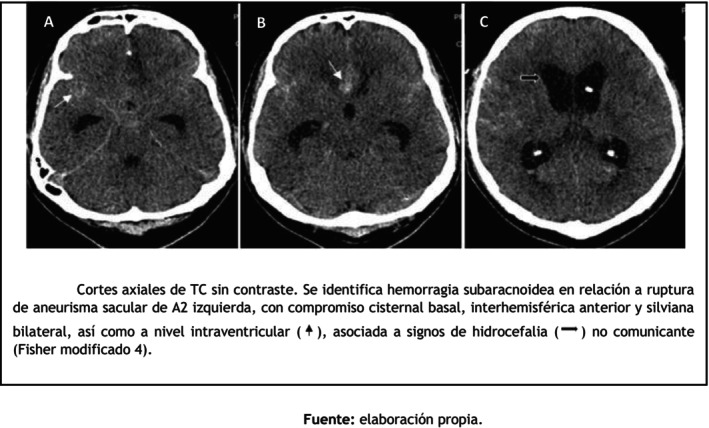
Non‐contrast axial brain CT images demonstrating subarachnoid hemorrhage secondary to rupture of a left A2 saccular aneurysm, with blood distribution involving the basal cisterns, anterior interhemispheric fissure, bilateral sylvian fissures, and intraventricular extension (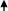), associated with signs of non‐communicating hydrocephalus (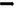) (modified Fisher grade 4). *Source:* Prepared by the authors.

On admission, neurological examination revealed a Glasgow Coma Scale (GCS) score of 14/15, corresponding to Hunt and Hess grade III and WFNS grade II based on the initial neurological examination. Non‐contrast computed tomography (CT) demonstrated subarachnoid hemorrhage consistent with modified Fisher grade 4 (Figure 1), secondary to rupture of a saccular aneurysm located at the bifurcation of the pericallosal and callosomarginal arteries of the left anterior cerebral artery, confirmed by digital subtraction angiography.

## 3 Investigations and Treatment

**FIGURE 2:**
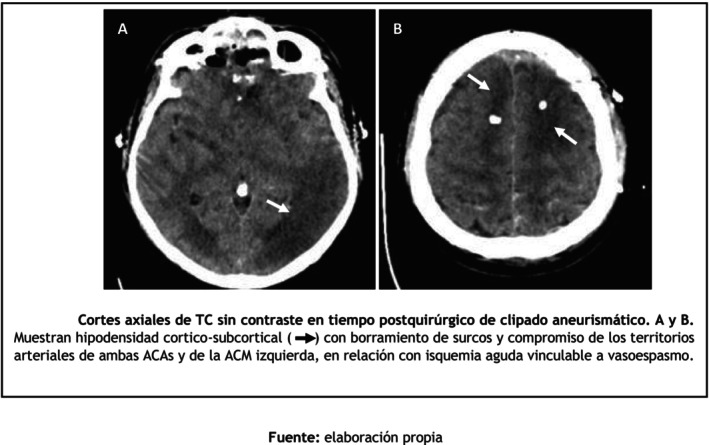
Postoperative non‐contrast axial brain CT images after aneurysm clipping. (A, B) Cortical‐subcortical hypodensity (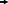) with sulcal effacement involving the vascular territories of both anterior cerebral arteries (ACAs) and the left middle cerebral artery (MCA), consistent with acute ischemia related to cerebral vasospasm. *Source:* Prepared by the authors.

**FIGURE 3:**
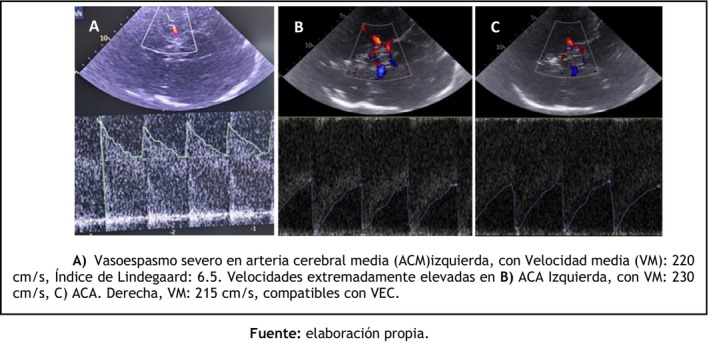
(A) Severe vasospasm of the left middle cerebral artery (MCA), with a mean flow velocity (MFV) of 220 cm/s and a Lindegaard Index of 6.5. Markedly elevated velocities were also observed in the (B) left anterior cerebral artery (ACA), with an MFV of 230 cm/s, and the (C) right ACA, with an MFV of 215 cm/s, findings consistent with severe cerebral vasospasm. *Source:* Prepared by the authors.

The patient underwent microsurgical clipping of the aneurysm on post‐bleed day 6. This strategy was preferred due to the presence of cerebrospinal fluid fistula secondary to cribriform plate injury, which increased the risk of infection with an endovascular approach. The procedure also included dural repair and placement of an external ventricular drain for the management of acute hydrocephalus and intracranial pressure control. In the immediate postoperative period, the patient was extubated with a GCS score of 13/15. However, 48 h later, he developed acute neurological deterioration, with a decline in GCS to 10/15, right‐sided hemiplegia, and impaired airway protection requiring reintubation. Emergency CT imaging demonstrated cortical hypodensities suggestive of delayed cerebral ischemia, while transcranial Doppler (TCD) revealed markedly elevated mean flow velocities consistent with severe vasospasm (Figures 2 and 3).

Protective mechanical ventilation was initiated, along with multimodal neuromonitoring, including intracranial pressure monitoring, capnography, serial TCD, pupillometry, and bispectral index monitoring. A bolus of intravenous milrinone (0.1 mg/kg) was administered, followed by continuous infusion starting at 0.75 μg/kg/min and titrated up to 1.5 μg/kg/min. Due to persistent severe vasospasm after 30 min, the patient was transferred to the angiography suite.

**FIGURE 4:**
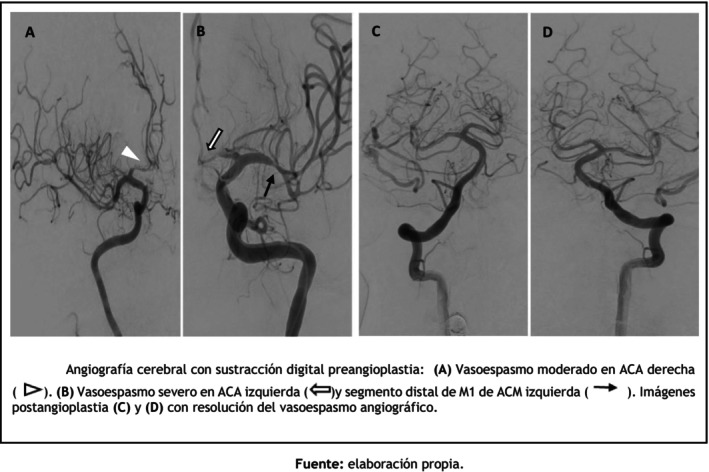
Digital subtraction cerebral angiography before angioplasty. (A) Moderate vasospasm of the right anterior cerebral artery (ACA) (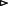). (B) Severe vasospasm of the left ACA (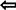) and the distal M1 segment of the left middle cerebral artery (MCA) (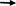). Post‐angioplasty images (C) and (D) demonstrate angiographic resolution of vasospasm. *Source:* Prepared by the authors.

Cerebral angiography confirmed severe bilateral vasospasm of the anterior cerebral arteries (A1 segments) and the left middle cerebral artery (M1 segment) (Figure 4A,B). Intra‐arterial milrinone was administered at a rate of 0.25 mg/min (total dose 10 mg). Due to persistent narrowing of the left anterior cerebral artery, rescue transluminal balloon angioplasty was performed, achieving a favorable angiographic response (Figure 4C,D).

Upon return to the intensive care unit, vasopressor support with norepinephrine (> 0.5 μg/kg/min) and vasopressin (0.04 IU/min) was initiated to maintain a mean arterial pressure ≥ 90 mmHg. Advanced hemodynamic monitoring using transpulmonary thermodilution was implemented, allowing individualized management of cardiac output, preload, and systemic vascular resistance.

## 4 Outcome and Follow‐Up

The clinical course was favorable, with progressive resolution of vasospasm confirmed by serial transcranial Doppler evaluations. Intravenous milrinone infusion was maintained for 4 days, facilitating sedation withdrawal, progressive neurological recovery, and successful extubation. At intensive care unit discharge, the patient had a GCS score of 13/15, right upper limb strength of 4/5 according to the Medical Research Council scale, and a modified Rankin Scale score of 3, with favorable prospects for functional recovery and social reintegration.

## 5 Discussion

Cerebral vasospasm (CVS) remains a major contributor to morbidity after aSAH, with progression to delayed cerebral ischemia (DCI) in approximately 30% of cases. Contemporary understanding recognizes DCI as a multifactorial syndrome rather than a purely macrovascular phenomenon, involving microcirculatory impairment, neuroinflammation, endothelial dysfunction, and cortical spreading depolarizations [8].

Our case illustrates the application of a modified Montreal protocol using high‐dose intravenous milrinone followed by intra‐arterial administration and selective balloon angioplasty. Previous reports have demonstrated that milrinone at supratherapeutic doses may reverse refractory vasospasm while reducing the need for rescue endovascular interventions [9, 10]. The MILRISPASM study further demonstrated a lower requirement for angioplasty in patients treated with milrinone compared with standard care [11].

Milrinone's pharmacological profile offers distinct advantages, combining selective cerebral vasodilation mediated by increased cyclic adenosine monophosphate with systemic inotropic support [6]. This contrasts with the transient effects and potential hemodynamic instability associated with other intra‐arterial vasodilators. Nonetheless, high‐dose milrinone frequently necessitates vasopressor support, underscoring the importance of comprehensive neuromonitoring to guide safe therapeutic escalation.

Beyond vasodilation, milrinone may exert pleiotropic effects, including improvement of cerebrovascular autoregulation, attenuation of neuroinflammation, and reduction of microthrombotic phenomena, potentially contributing to its efficacy even in the absence of critical angiographic vasospasm [12]. However, optimal timing, dosing strategies, and predictive biomarkers remain to be defined.

In resource‐limited settings, this case demonstrates that a physiology‐guided, escalation‐based protocol supported by neuromonitoring can be safely implemented, offering a viable option for managing refractory DCI.

This report has limitations. As a single‐case observation, its findings may not be generalizable, and no standardized biomarkers were used to predict response to milrinone. Further prospective studies are required to define optimal patient selection, dosing strategies, and timing of intervention.

## 6 Conclusion

This case supports a stepwise approach to refractory cerebral vasospasm after aSAH, in which high‐dose intravenous milrinone may serve as first‐line rescue therapy, intra‐arterial administration provides therapeutic escalation, and balloon angioplasty is reserved for non‐responders. Such a strategy acknowledges the multifactorial nature of DCI and emphasizes the critical role of intensive neuromonitoring in guiding safe and effective therapeutic escalation.

## Author Contributions

**Paul Cardozo Gil:** conceptualization, investigation, methodology, validation, visualization, writing – original draft, writing – review and editing. **Willy Pozo Soto:** conceptualization, investigation, writing – review and editing. **Alejandro Sengoku Cadima:** methodology, supervision, writing – review and editing. **Pablo Flores:** investigation, supervision. **Jorge Botello Marin:** conceptualization, investigation, visualization. **Gustavo Averanga:** conceptualization, validation, visualization. **Richy Hurtado Montaño:** supervision, validation, visualization. **Nelson Montalvo Flores:** methodology, writing – original draft.

## Funding

The authors have nothing to report.

## Consent

Written informed consent was obtained from the patient for publication of this case report.

## Conflicts of Interest

The authors declare no conflicts of interest.

## Data Availability

Data will be provided by the corresponding author upon reasonable request.
